# Sectional Anatomy Quiz - V

**DOI:** 10.22038/aojnmb.2019.44526.1301

**Published:** 2020

**Authors:** Rashid Hashmi

**Affiliations:** Rural Medical School, University of New South Wales (UNSW), Wagga Wagga, NSW, Australia

**Keywords:** Sectional anatomy, Computed tomography, Lung, Pulmonary fissures

## Abstract

In this series a pictorial quiz pertaining to identification of normal anatomical structures and landmarks at a given level on the computed tomography (CT) is presented. An image depicting normal anatomy is followed by a series of images showing different pathologies. Readers are expected to identify and appreciate variation and changes in the normal anatomy in presence of a given pathology. The series is intended to enhance understanding of sectional anatomy thus aiding interpretation of the CT component of the single photon emission computed tomography (SPECT) and positron emission tomography (PET) studies.

## Introduction

**Figure 1:**
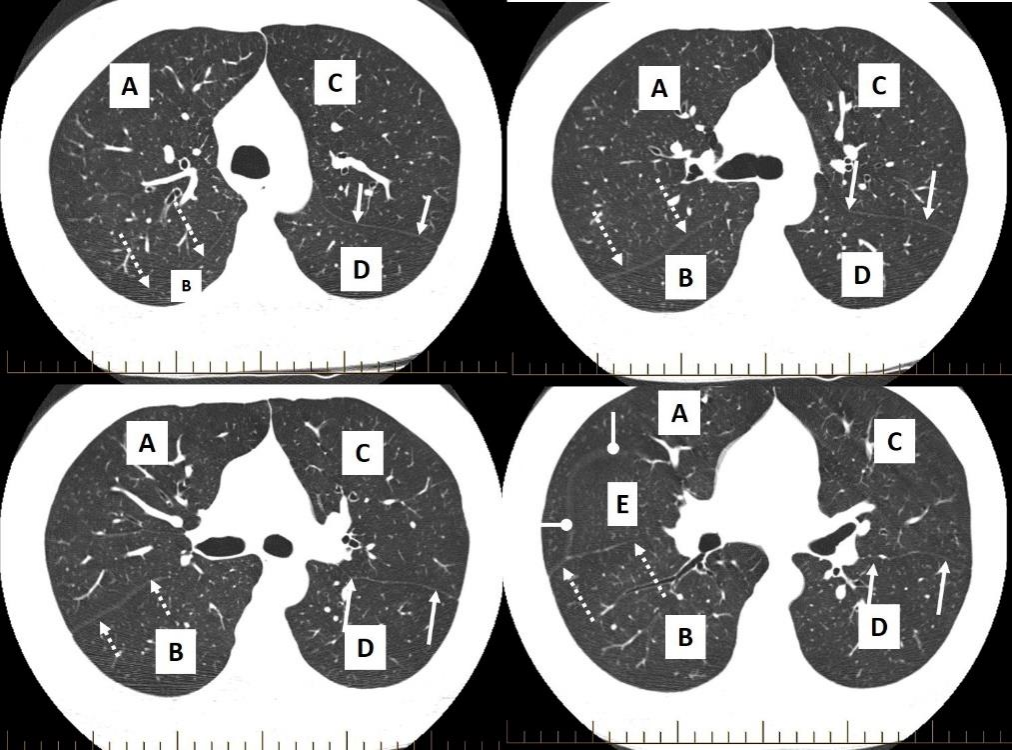
Multiple axial CT images (lung window) of the chest of a 51 years old man are shown. Identify the structures depicted as white lines and marked by dotted, solid and oval arrows in both the lungs. Also name the lobes in both the lungs labelled A to E

**Answer**

 The images show normal lung parenchyma. Top left and right images are acquired slightly above and at the level of the carina (tracheal bifurcation) respectively while bottom left and right images are below the level of the carina. 

 White lines marked as dotted, solid and oval arrows in both the lungs are right oblique (Major) fissure, left oblique fissure and right horizontal 

(Minor) fissure respectively. Note relative absence of vascular marking along the fissures. This is particularly marked along the horizontal fissure. 

A: Right upper lobe

B: Right lower lobe

C: Left upper lobe

D: Left lower lobe

E: Right middle lobe

**Figure 2:**
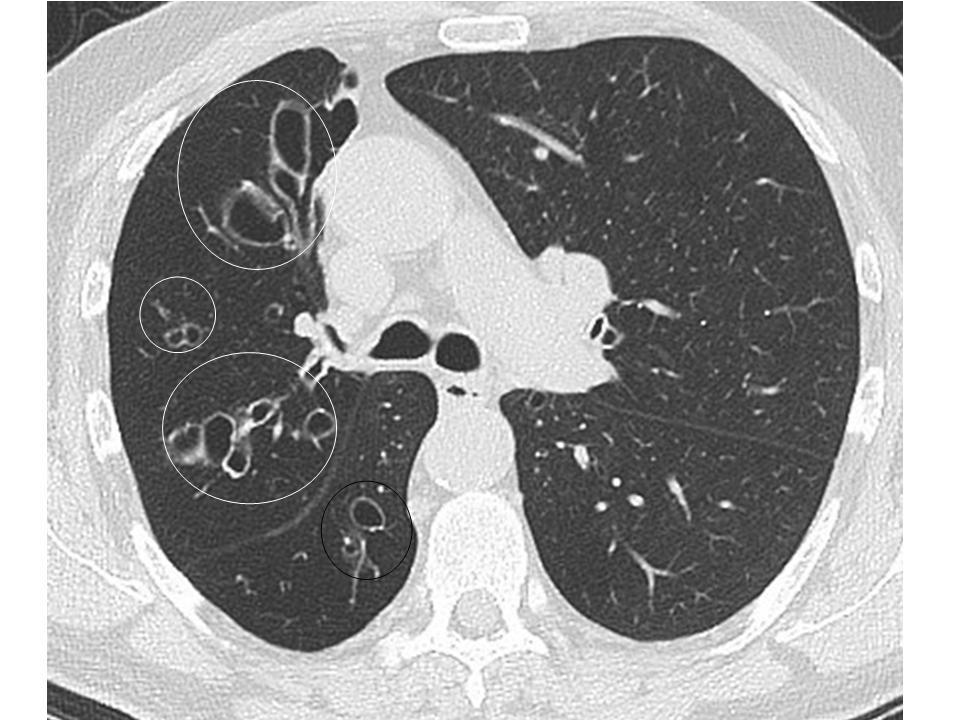
An axial CT image (lung window) of the chest of a 64 years old male with known bronchiectasis shows multiple cluster of cystic areas in the upper (white circles) and lower (black circle) lobes of the right lung. Oblique fissures are well outlined in both the lungs

**Figure 3:**
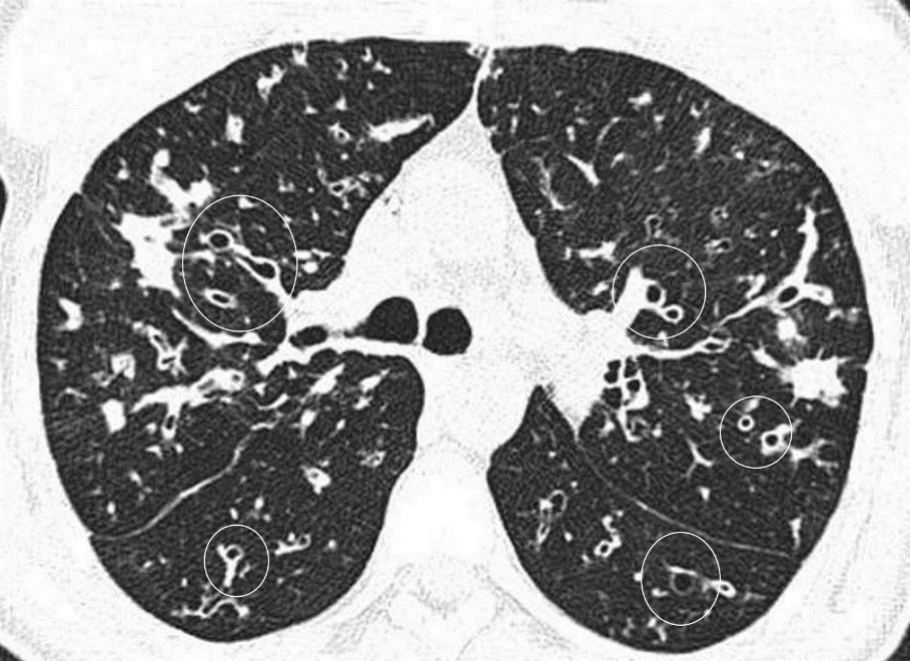
An axial CT image (lung window) of the chest of a 24 years old female with history of cystic fibrosis shows multiple irregular opacities and cystic changes in both the lungs. Cystic changes (circled) are suggestive of bronchiectasis. Note the thickened wall of dilated bronchi. Right oblique fissure is also mildly thickened

**Figure 4:**
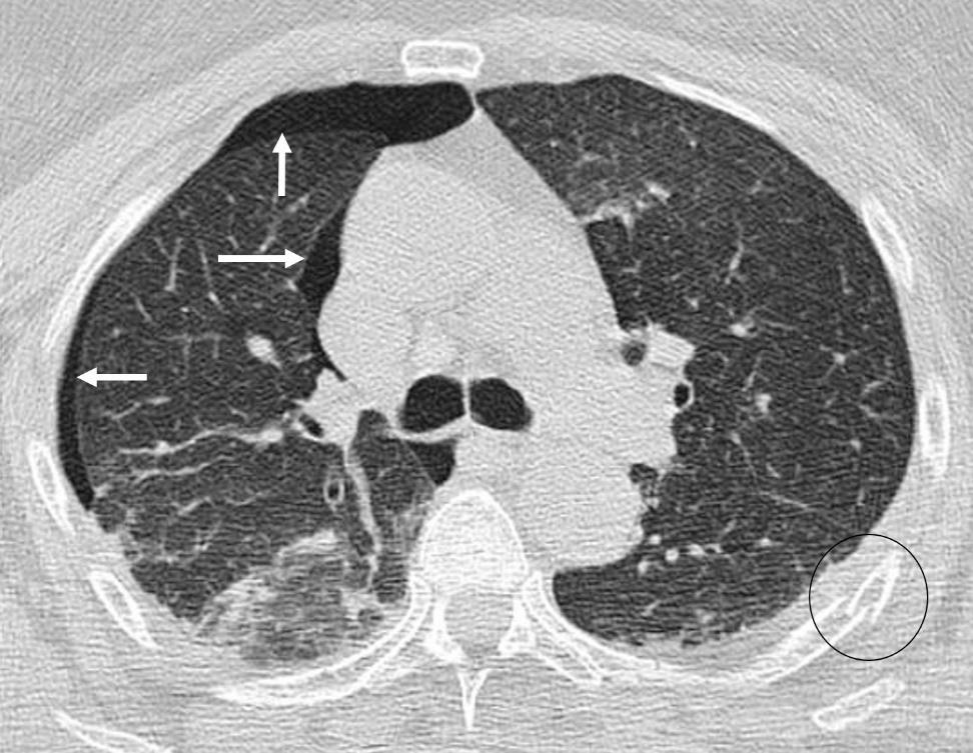
An axial CT image (lung window) of the chest of 21 years old man obtained after a high-speed car crash shows right sided pneumothorax. Solid arrows point to the margin of the displaced visceral pleura. Right oblique fissure is not clearly delineated and is obscured by the ground glass opacities in the posterior aspect of the right lung which given it location is in the right lower lobe and suggestive of pulmonary contusion. Patient had multiple rib fractures bilaterally but only a left sided fracture (circled) is seen on this image. It shall be noted that fractures and other skeletal pathologies are best visible on the bone window

**Figure 5:**
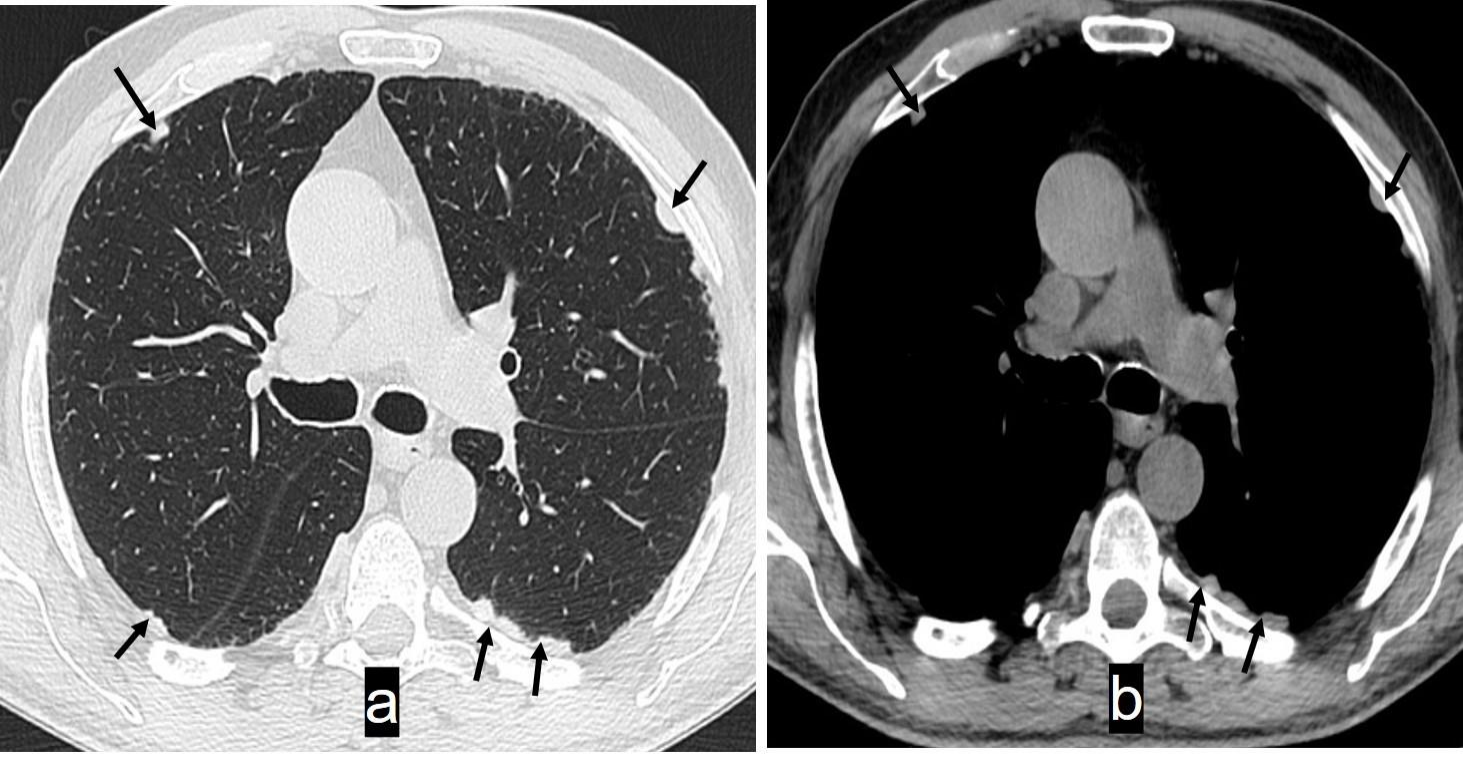
Axial non-contrast CT images of the chest of a 65 years old builder with history of exposure to asbestos show multiple pleural plaques (solid arrows) bilaterally. Plaques are appreciated on the lung (a) and mediastinal (b) windows but their density and size are better delineated on the mediastinal window

Readers shall identify the normal vascular anatomy of the mediastinum at this level and, for review, can refer to the first article of this series (1).

**Figure 6:**
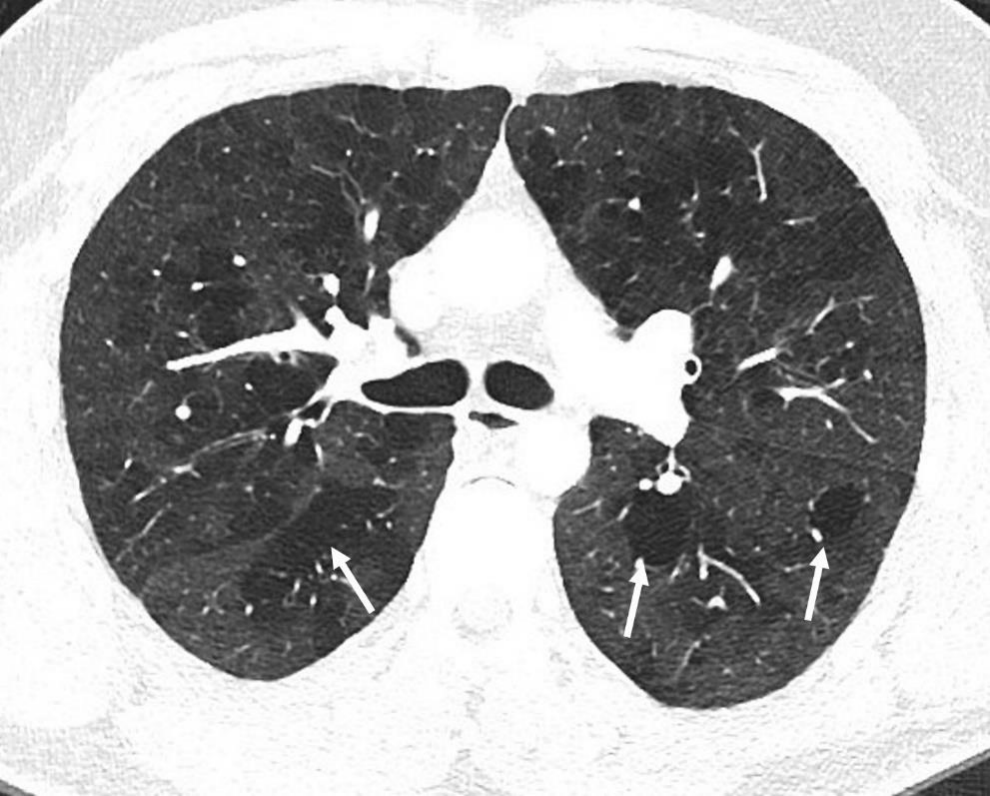
An axil CT image (lung window) of the chest of a 54 years old male smoker shows widespread emphysematous changes in both the lungs. These changes appear as poorly marginated lucent areas of varying sizes within the lung parenchyma (“holes in lung”). A few of these areas in bilateral lower lobes are marked (solid arrows). Right oblique fissure is not delineated on the image

**Figure 7:**
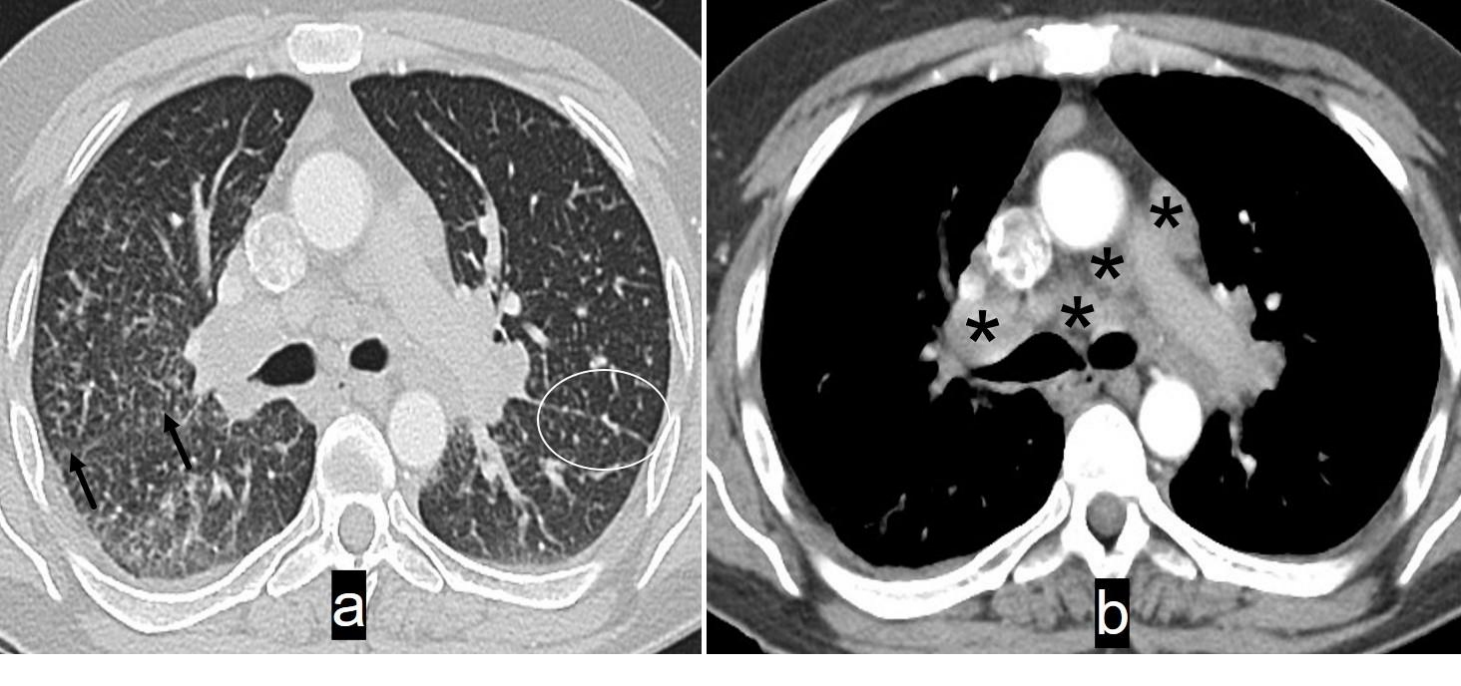
Contrast enhanced CT of a 31 years old female with sarcoidosis. Lung window (a) shows widespread reticulonodular opacities in both the lungs. Note presence of small nodules along the left oblique fissure (circled) suggesting peri-lymphatic distribution of nodules - a hallmark of sarcoidosis. Right oblique fissure is largely obscured by the opacities, but its probable location is indicated (arrows). Mediastinal window (b) shows multiple enlarged lymph nodes (asterisks) in the pre-carinal, pre-vascular and right hilar regions

**Figure 8:**
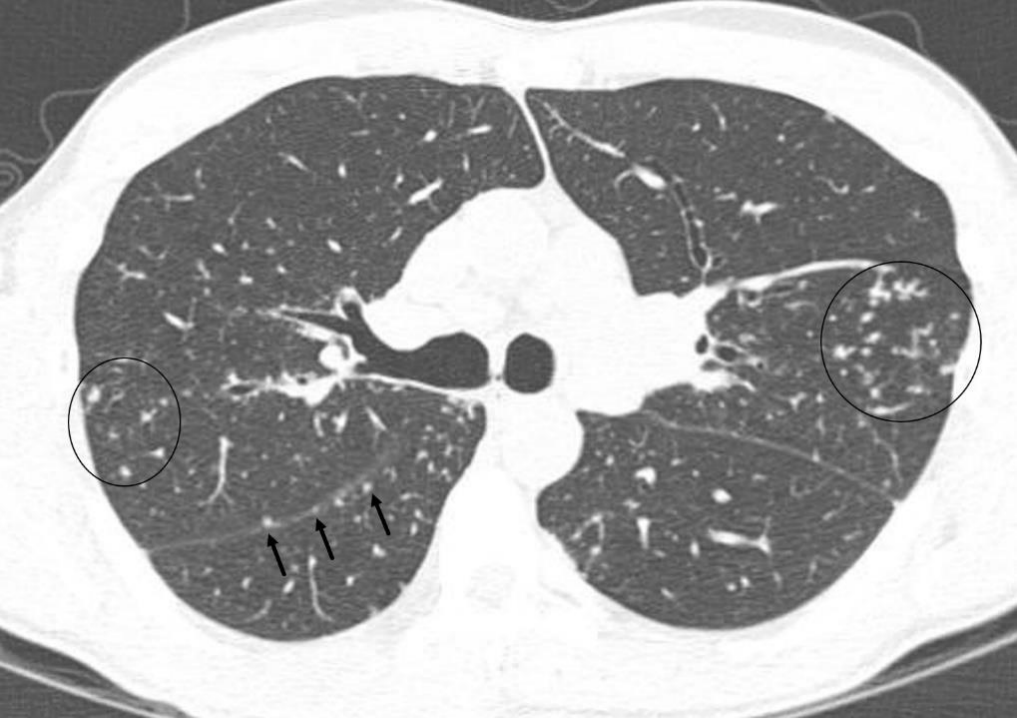
An axial CT image (lung window) of a 41 years old female with sarcoidosis shows cluster of nodular and branching opacities (circled) in the lateral aspect of upper lobes of both the lungs. A few of these opacities abut the pleural surface of lung. Note presence of small nodules along the oblique fissures particularly pronounced in the right lung (arrows).

**Figure 9:**
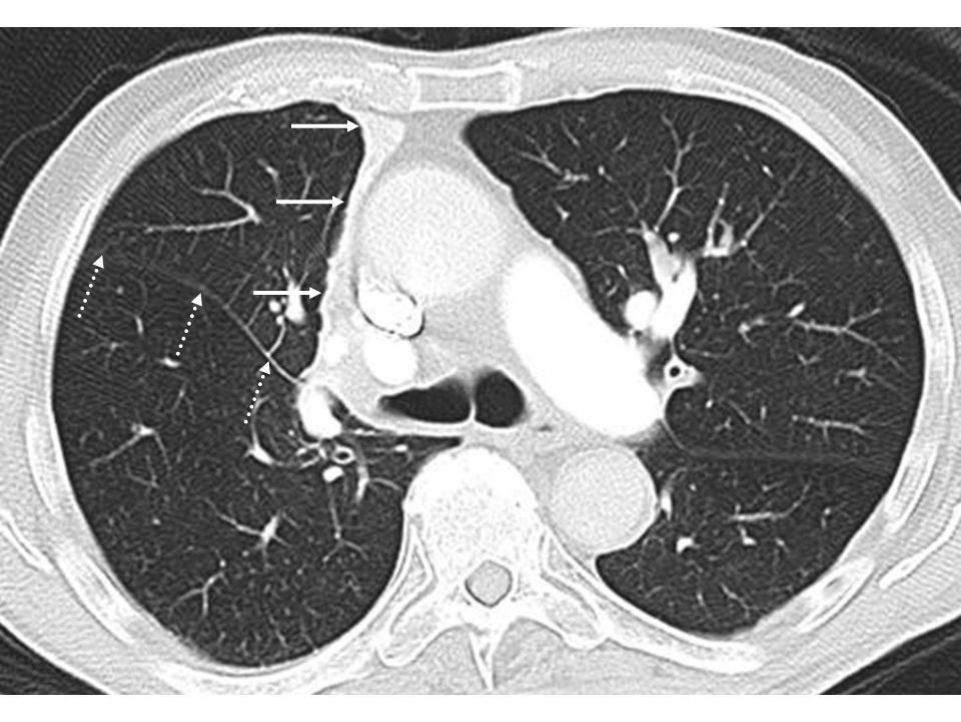
An axial CT image (lung window) of a 62 years old smoker with partial collapse of the right upper lobe secondary to a bronchogenic carcinoma shows anterior displacement of the right oblique fissure (dotted arrows). Solid arrows point to the lateral margin of the collapsed segment of the right upper lobe. Bronchogenic carcinoma is not seen on this image. Left oblique fissure is normal in its position

**Figure 10:**
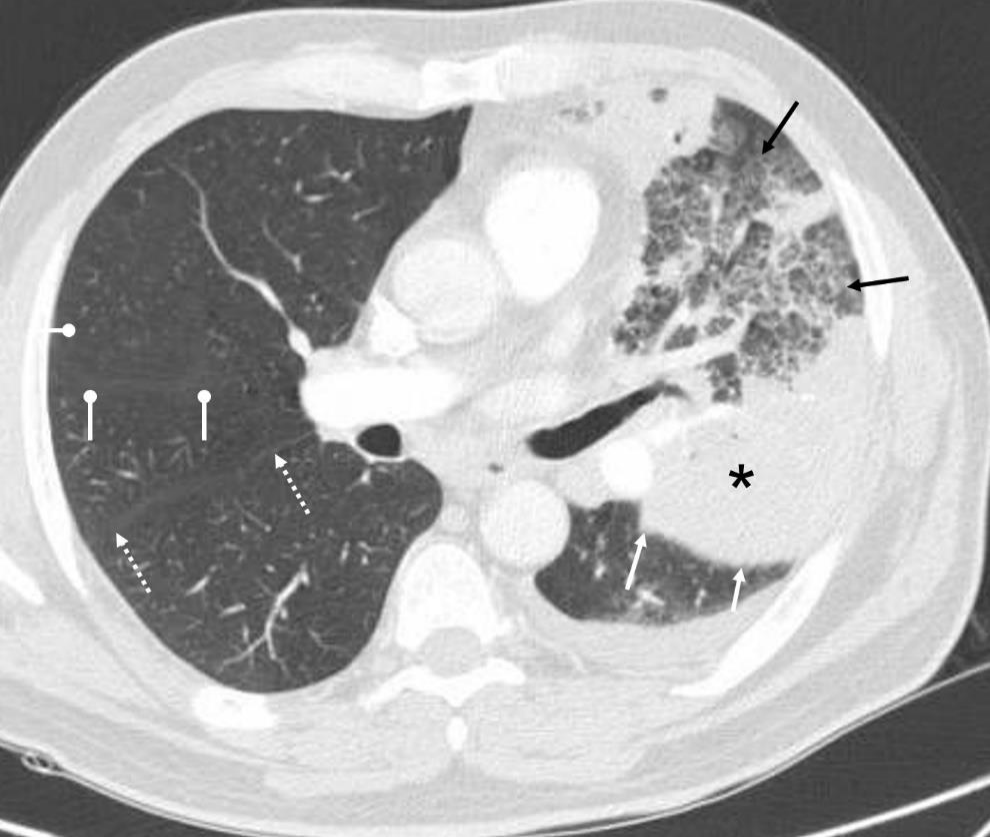
An axial CT image (lung window) of a 71 years old female with pneumonia shows a large area of homogeneously increased density suggesting consolidation (asterisk) and ill-defined heterogeneous areas of increased density suggesting glass opacities (solid black arrows) in the left upper lobe

On CT, consolidation refers to an area of abnormality that appears as homogenous increase in lung parenchymal attenuation that obscures the margins of the vessels and airways. Ground glass opacity, conversely, refers to an area of abnormality that appears as increase in lung parenchymal attenuation with preservation of vessels and airways. It is important to remember that consolidation and ground glass opacity are descriptive terminologies and can be manifestation of a wide range of pulmonary pathologies including pneumonia.

Convexity along the posterior margin of the consolidation (solid white arrows) represents the left oblique fissure. Oblique and horizontal fissures in the right lung are marked by dotted and oval arrows respectively.

**POINTS TO REMEMBER**

 - Pulmonary fissures are double fold of visceral pleura that invaginate the lung parenchyma completely or incompletely to form lobes. Each lung has an oblique fissure (variably called major or greater fissure) separating the upper and lower lobes. Additionally, right lung has a horizontal fissure (variably called minor or transverse fissure) separating the upper lobe from the middle lobe. 

 - Oblique fissures extend from the level of 4^th^/5^th^ thoracic vertebra posteriorly to the diaphragm anteriorly and inferiorly. Medial aspect of both the fissures passes through the respective hila. Due to their undulating course, fissures do not follow a straight path from top to bottom. 

 - Horizontal fissure runs horizontally from the hilum to the anterior and lateral aspect of the right lung at the level of the 4^th^ costal cartilage. Its posterior limit is the right oblique fissure, which it meets at the level of the 6^th^ rib in the mid axillary line. It is highly variable and can be incomplete or absent in many normal individuals. 

- On CT, the oblique fissures are oriented obliquely to the scan plane and have variable appearance which is also dependent on the slice thickness. On high resolution CT, the fissures appear as a sharp thin white line surrounded by a band of avascular lung. On conventional CT, the fissures are visible in approximately 40% of cases as an ill-defined band of opacity. However, their position can often be inferred by the presence of an avascular band within the lung parenchyma. 

 - The orientation of the oblique fissures varies at different levels in the thorax. In the upper thorax, the fissures angle posterolaterally from the mediastinum. In the lower thorax, the fissures angle anterolaterally from the mediastinum.

 - Horizontal fissure is more variable in appearance on the CT. Depending on its orientation and contour, on high resolution CT, it can be visible as i ) an ill-defined opacity, ii) a band of avascular region, iii) a thin white line directed from anterior to posterior or iv) a thin white line extending medial to lateral parallel and anterior to the right major fissure. On conventional CT, horizontal fissure is rarely visible, but its position can be inferred by an avascular region anterior to the right oblique fissure below the level of carina.

**Recommendations for Further Reading**

1. Hayashi K, Aziz A, Ashizawa K, Hayashi H, Nagaoki K, Otsuji H. Radiographic and CT appearances of the major fissures. RadioGraphics. 2001; 21(4):861-74. 

2. Aziz A, Ashizawa K, Nagaoki K, Hayashi K. High resolution CT anatomy of the pulmonary fissures. J Thorac Imaging. 2004; 19(3):186-91.

3. Ryan S, McNicholas M, Eustace S. Anatomy for Diagnostic Imaging. 3rd Edition. Elsevier. 2010. 

4. Currie S, Kennish S, Flood K. Essential Radiological Anatomy for the MRCS. Cambridge University Press. 2009. 

5. Moeller T, Reif E. Pocket Atlas of Sectional Anatomy. Computed Tomography and Magnetic Resonance Imaging. Volume 2. Thorax, Abdomen, and Pelvis. Thieme Stuttgart. 2001.
